# Associations between social network addiction, anxiety symptoms, and risk of metabolic syndrome in Peruvian adolescents—a cross-sectional study

**DOI:** 10.3389/fpubh.2024.1261133

**Published:** 2024-05-01

**Authors:** Jacksaint Saintila, Susan M. Oblitas-Guerrero, Giovanna Larrain-Tavara, Isabel G. Lizarraga-De-Maguiña, Fátima del Carmen Bernal-Corrales, Elmer López-López, Yaquelin E. Calizaya-Milla, Antonio Serpa-Barrientos, Cristian Ramos-Vera

**Affiliations:** ^1^Escuela de Medicina Humana, Universidad Señor de Sipán, Chiclayo, Peru; ^2^Escuela de Enfermería, Universidad Señor de Sipán, Chiclayo, Peru; ^3^Research Group for Nutrition and Lifestyle, Universidad Peruana Unión, Lima, Peru; ^4^Departamento de Psicología, Universidad Nacional Mayor de San Marcos, Lima, Peru; ^5^Area de Investigación, Universidad Cesar Vallejo (UCV), Lima, Peru

**Keywords:** adolescents, anxiety symptoms, cardiometabolic risk, metabolic syndrome, obesity, social network addiction

## Abstract

**Background:**

The link between physical and mental health and screen time in adolescents has been the subject of scientific scrutiny in recent years. However, there are few studies that have evaluated the association between social network addiction (SNA) and metabolic risk in this population.

**Objective:**

This study determined the association between SNA and anxiety symptoms with the risk of metabolic syndrome (MetS) in adolescents.

**Methods:**

A cross-sectional study was conducted in Peruvian adolescents aged 12 to 18 years, who completed a Social Network Addiction Questionnaire and the Generalized Anxiety Disorder 2-item scale (GAD-2), between September and November 2022. A total of 903 participants were included in the study using a non-probability convenience sample. Sociodemographic and anthropometric data were also collected. Binary logistic regression was used to explore the association between SNA and anxiety symptoms with MetS in a cross-sectional analysis.

**Results:**

Males were more likely to have MetS than females (OR = 1.133, *p* = 0.028). Participants who were 16 years of age or older and those with excess body weight were 2.166, *p* = 0.013 and 19.414, *p* < 0.001 times more likely to have MetS, respectively. Additionally, SNA (OR = 1.517, *p* = 0.016) and the presence of anxiety symptoms (OR = 2.596, *p* < 0.001) were associated with MetS.

**Conclusion:**

Our findings suggest associations between SNA, anxiety symptoms, and MetS among youth. However, more studies are needed to better understand this association and to deepen the possible clinical and public health implications.

## Introduction

Metabolic syndrome (MetS) also known as “insulin resistance syndrome” or “syndrome X” refers to the set of conditions that increase the likelihood of developing cardiovascular and metabolic diseases, including type 2 diabetes mellitus, hypertension, and cardiovascular disease (1). Central obesity, which can be measured through anthropometric parameters, such as waist and hip circumferences and waist-hip ratio (WHR), constitutes one of the main components of the MetS (2). In recent decades, the prevalence of obesity in adolescents has increased significantly worldwide (3), which has been associated with an increased risk of developing cardiometabolic diseases at an early age (4). In fact, since 1975, obesity rates have increased considerably and have almost tripled in the general population, and in the particular case of children and adolescents, this increase is even greater, reaching almost a five-fold increase (3). In the national context, in recent years, there has been a steady increase in the number of Peruvian adolescents at high and very high risk of cardiometabolic diseases, as measured by the WHtR (5), which, in turn, increases mortality risk. In Peru, there is a double burden of morbidity due to, on the one hand, problems associated with infectious diseases and malnutrition by deficit and/or excess, and on the other hand, a progressive increase in non-communicable diseases (6).

Social networks are online platforms that allow users to create personal profiles and establish connections with other users in their network (7). In recent years, the use of social networks has become an increasingly frequent and popular activity among adolescents (8). Social network platforms such as TikTok, Facebook, Twitter, among others, can represent a valuable opportunity to connect and engage adolescents with messages related to the adoption of healthy lifestyles, such as proper diet and physical activity, which can be a preventive element against MetS (9). However, excessive exposure to these digital platforms could have negative consequences on the physical and mental health of this population group (8). There are few studies that examine the relationship between SNA and MetS. However, we found a recent study that evaluated the link between adolescent social networks and health in adulthood, suggesting that adolescents’ social network position has lasting consequences for MetS in adulthood (10). However, most studies have focused on body mass index (BMI), pointing to a possible relationship between social media addiction, excessive use of media, and an increased risk of developing obesity in adolescents (11–13), which is related to cardiometabolic problems (1, 4).

Anxiety symptoms are one of the most prevalent psychiatric conditions in adolescence, affecting approximately 1 in 4 adolescents (14). These symptoms may represent risk factors for anxiety disorders encompassing various conditions such as agoraphobia, panic disorder, specific phobias, separation anxiety disorder, social anxiety disorder, and generalized anxiety disorder (15). Studies have shown that anxiety symptoms are associated with an increased risk of cardiovascular disease and other metabolic problems in young adults (16, 17). Although there is less research examining these connections in adolescents, a study recently found a statistically significant relationship between anxiety and some metabolic risk factors in this population (18). Another study conducted in adolescents reported a relationship between anxiety symptoms and insulin resistance (19), which may lead to worsening metabolic outcomes in at-risk youth.

Therefore, considering the above, it is important to deepen our understanding of this relationship and explore the possible clinical and public health implications. In this context, the present study aims to examine the association between SNA, anxiety symptoms, and MetS in adolescents, with the intention of contributing to a better understanding of these phenomena and to the implementation of effective preventive and therapeutic interventions.

## Materials and methods

### Design and study participants

A descriptive cross-sectional study was conducted during September and November 2022. The sample was selected using non-probabilistic sampling (20, 21). The researchers chose to use non-probabilistic purpose sampling because it is relevant to obtain data from respondents for this study (21–23). Data were collected using a survey consisting of the following: (a) sociodemographic data (e.g., age, sex, origin, place of residence, among others); (b) a validated questionnaire and scale to assess symptoms of SNA and anxiety symptoms, respectively; (c) in addition, information was collected on body weight status, height, and waist circumference, to subsequently estimate BMI, height-for-age (H/A), and waist-to-height ratio (WHtR).

The survey was distributed to participants enrolled in two public schools in the districts of Reque and Morrope, located in the city of Chiclayo, Peru. Data collection was possible due to the support of the directors of both schools and the teachers of each of the classrooms we selected. The sample size was calculated using Free Statistic Calculators version 4.0 (24). For the multiple regression analysis, an effect size of 0.10, a statistical power of 0.80, 5 explanatory variables and a probability level of 0.05 were considered. According to this calculation, a minimum sample size of 134 participants was required. However, in this study, a total of 903 students participated voluntarily, which far exceeds the calculated sample size. Participants of both sexes, those without any pathology, and those within the selected age range (12–18 years) were included. However, adolescents whose parents did not give their written informed consent were excluded from the study. Furthermore, 23 records were excluded due to missing data. The final sample was 903 participants.

### Ethical aspects

The study was carried out after receiving the approval of the Research Ethics Committee of the Universidad Señor de Sipán (Registration and reference number: 0085-17052022-CIEI). Subsequently, the directors of both schools were contacted to request and obtain permission to meet with the parents of potential participants. This meeting was to explain to all parents the purpose of the study. Furthermore, considering that the participants were minors, a procedure was implemented to guarantee participation with the consent of the parents or legal guardians. After providing initial explanations about the purpose of the study, an informed consent question was included to be answered by parents or legal guardians as a prerequisite for adolescents to participate in the survey. Therefore, informed consent was obtained from all subjects prior to their participation in the study.

### Measurement instruments

#### Social network addiction questionnaire

This instrument was originally developed by Escurra and Salas in 2014 and was constructed using a sample of 380 participants (36.3% men and 63.7% women) in Lima, Peru (25). This questionnaire is made up of 24 items on a 5-point Likert scale ranging from never to always (assigned scores from 0 to 4). In addition, it has 23 direct items and 1 inverse item. A higher score indicates a higher level of SNA. The validity and reliability of the instrument was analyzed, showing a Cronbach’s Alpha coefficient (α) = 0.95, therefore, it has adequate internal consistency. Additionally, the instrument evaluation was carried out on a sample of 744 adolescents aged 17 to 19 years, reporting a reliability of α = 0.86 (26). In the current study, the internal consistency of the instrument was also tested, evidencing α = 0.89.

#### Generalized anxiety disorder (GAD-2)

The Generalized Anxiety Disorder 2-item Scale was used to measure emotional state. This instrument is composed of item 1 of the GAD-7 “Feeling of nervousness, anxiety, or being on edge” and by item 2 “Not being able to stop worrying or controlling worries” and is assessed through the question: “Indicate how often you have experienced the following problems in the last 15 days” (27, 28). These items have 4 response options where never = 0, less than half of the days = 1, more than half of the days = 2, and almost every day = 3. A cutoff score greater than or equal to 3 on GAD-2 is an indicator of a probable clinically relevant anxiety disorder, while a score less than 3 indicates the absence of anxiety symptoms (29). Total scores range from 0 to 6 (28, 30, 31). In this study, the version adapted and validated for the Peruvian population was used and presented an adequate Cronbach’s α coefficient (α = 0.81) (32).

#### Sociodemographic data

Sociodemographic and economic data were collected through a registration form, which is composed of sociodemographic factors such as age in years (11–12 and 16–18), sex (male and female), residence (urban and rural), level of education of parents (elementary, technical, and university), marital status of parents (married, cohabiting, single, divorced, and widowed), family income in “soles (PEN)” (<2,149.00 PEN, 2,149.00 PEN–10,746.00 PEN, and > 10,746.00 PEN), among others.

### Anthropometric data

#### BMI

Weight and height were measured using a calibrated SECA 700 mechanical column scale with a capacity of 220 kg and a measuring range of 60 to 200 cm (SECA®, Hamburg, Germany). Anthropometric evaluation was performed by a professional nutritionist in the early hours of the day for one week. Furthermore, the measurements were performed with the participants walking barefoot and wearing the minimum amount of clothing. The BMI was calculated, and the classification was made according to the parameters established by the World Health Organization. A BMI z score was determined and classified as follows: “underweight,” BMI z-score < −1; “normal,” BMI z score −1 to 1; and “overweight” (z > 1) (33).

#### Height/age (H/A)

Furthermore, H / A was calculated and classified based on the reference data corresponding to the Peruvian standards in the public health system: >2 standard deviation (SD), “normal or adequate” (H/A ≥ −2 to ≤2 SD), “low” (H/A < −2 to −3 SD), and “severe low” (H/A < −3). For the purposes of the current study, it is necessary to specify that short and very severe height was recategorized as short height (34, 35).

#### Waist-to-height ratio (WHtR)

Waist circumference (WC) was measured in triplicate using a Cescorf (Cescorf Equipamentos Para Esporte Ltda—Epp, Brazil) self-retractable metallic steel tape measure. Measurement of the WC was made considering the midpoint of the axillary line, in the distance that goes from the ridge of the last rib to the iliac spine (36).

### Outcome

After obtaining WC measurements, WHtR was determined by dividing the waist circumference of each participant by their respective height. This anthropometric parameter is valued for its ability to provide an accurate indication of the distribution of adipose tissue in the body (33, 37). It is a simple index that offers immediate identification and interpretation, being particularly useful in the early identification of abdominal obesity in children (38). As a result, it makes it possible to anticipate potential risks related to cardiometabolic disorders (2, 37, 39). In the evaluation process, a cutoff point of 0.5 was identified. That is, participants who had a WHtR index greater than 0.5 were classified as adolescents at risk of MetS (2, 33, 37, 39, 40).

### Statistical analysis

A Microsoft Excel spreadsheet was used for data collection and coding. Then, for data processing and analysis, the IBM SPSS statistical software package, version 26 (SPSS Inc., Chicago, IL, United States) was used. Descriptive analysis of the variables was performed using tables of absolute frequencies and percentages. To explore whether sociodemographic data, anthropometric data, SNA, and anxiety symptoms were different according to sex and MetS, the chi-square statistical test was used. Finally, an exploration of the association of the factors influencing MetS (dependent variable) was carried out using a binary logistic regression model. We considered sex, age, overweight, SNA, and anxiety symptoms as independent variables. These variables had a probability value (*p*-value) of less than 0.05 in a preliminary bivariate analysis, and therefore were incorporated in the bivariate logistic regression analysis.

## Results

A total of 903 schoolchildren voluntarily decided to participate in the study; of these, 56% were female. The highest proportion (68.8%) were between 12 and 15 years of age. Regarding the level of education of parents, 76.6% of mothers and 69.2% of fathers reported basic education. Most parents were married (46.4%), had a monthly income <2,149.00 (74.6%), and reported having more than two sons (57.9%). More than half of the respondents reported that they live with their parents (55.3%). The sociodemographic characteristics of the participants are shown in Table 1.

**Table 1:** Sociodemographic characteristics of the participants (*N* = 903).

Variable		*n*	%
Sex	Female	497	55.0
	Male	406	45.0
Age	12–15	621	68.8
	16–18	282	31.2
Mother’s education	None	107	11.8
	Basic	692	76.6
	Technical	67	7.4
	University	36	4.0
Father’s education	None	109	12.1
	Basic	625	69.2
	Technical	92	10.2
	University	77	8.5
Parents’ marital status	Married	419	46.4
	Cohabitant	208	23.0
	Single	139	15.4
	Divorced	105	11.6
	Widowed	31	3.4
With whom you live	With mother and father	499	55.3
	Only with father	94	10.4
	Only with mother	201	22.3
	With another family member	103	12.2
Monthly income	<2,149.00 PEN*	674	74.6
	2,149.00–10,746.00 PEN	168	18.6
	>10,746.00 PEN	60	6.6
Number of children	One son	140	15.5
	Two sons	240	26.6
	More than two sons	523	57.9

*The ISO code for Peruvian currency is PEN, a standardized 3-letter code according to the ISO-4217 currency code standard.

The results of the association between H/A, BMI, SNA, and anxiety symptoms are shown in Table 2. MetS was generally observed among men (67.1%, *p* = 0.046), in the age range of 16–18 (78.3%, *p* < 0.001), in those with excess body weight (90.1%, *p* < 0.001), SNA (85.5%, *p* = 0.039) and anxiety symptoms (63.2%, *p* = 0.007).

**Table 2:** Chi-square analysis of MetS.

Variables	MetS		Non MetS			
	*n*	%	*n*	%	χ^2^	*p*-value
Sex						
Female	50	32.9	436	58.1	0.421	0.046*
Male	102	67.1	315	41.9		
Age (years)					15.440	<0.001*
12–15	33	21.7	412	54.8		
16–18	119	78.3	339	45.2		
H/A						
Inadequate H/A	52	34.2	286	38.1	0.809	0.368
Adequate H/A	100	65.8	465	61.9		
BMI						
Normal	15	9.9	479	63.8	148.290	<0.001*
Excess body weight	137	90.1	272	36.2		
SNA						
Yes	130	85.5	90	12.0	0.721	0.039*
No	22	14.5	661	88.0		
Anxiety symptoms						
Yes	96	63.2	246	32.8	0.948	0.007*
No	56	36.8	505	67.2		

*Statistically significant, *p* < 0.05 [Chi-square (χ^2^)]. *p* represents the probability that MetS is associated with sociodemographic and anthropometric data, social network addiction, and anxiety symptoms. H/A, height/age; BMI, body mass index; MetS, Metabolic syndrome; SNA, Addiction to social networks.

A binary logistic regression model was used to explore the variables that predict the probability that adolescents will present MetS and the results are shown in Table 3. In this analysis, males were more likely to present MetS than females (OR = 1.133, *p* = 0.028). Participants who were 16 years of age or older and those with excess body weight were 2.166, *p* = 0.013 and 19.414, *p* < 0.001 times more likely to have MetS, respectively. Furthermore, SNA (OR = 1.517, *p* = 0.016) and the presence of anxiety symptoms (OR = 2.596, *p* < 0.001) were associated with the risk of MetS.

**Table 3:** Binary logistic regression analysis of factors associated with MetS.

Variables				95% CI	
	*B*	OR_B_	*p*	Lower	Upper
Sex (0 = female, 1 = male)	0.125	1.133	0.028	0.770	1.667
Age (year) (0 = <16, 1 = ≥16)	0.773	2.166	0.013	1.386	3.387
Excess body weight (0 = no, 1 = yes)	2.966	19.414	<0.001	10.911	34.544
SNA (0 = no, 1 = yes)	0.417	1.517	0.016	0.844	2.727
Anxiety symptoms (0 = no, 1 = yes)	0.954	2.596	<0.001	1.713	3.933

χ^2^ = 173,114, df = 5, *p* < 0.001; Cox and Snell R-squared = 0.174, Nagelkerke R-squared = 0.293.

B, Beta coefficient; *p*, probability; OR_B_, Odds ratio; 95% CI, 95% confidence interval; SNA, social network addiction.

## Discussion

In this cross-sectional study, we determined the association between SNA and anxiety symptoms with the risk of MetS in Peruvian adolescents aged 12 to 18 years. The main findings were as follows: Male sex, participants who were 16 years of age or older and those who had excess body weight were more likely to present MetS. Furthermore, it is highlighted that SNA and anxiety symptoms were associated with MetS.

Previous studies have documented the influence of sex and age on the anthropometric profile, more precisely MetS, measured through the WHtR (4, 41–43). The results of the logistic regression analysis of the current study revealed that men were more likely to have MetS compared to females; furthermore, we found that a higher proportion of men had MetS. It should be noted that the current study used WHtR to determine MetS. Similarly, the findings of the research conducted in Brazilian adolescents showed that boys had a higher mean WHtR and a higher WHtR at the 95^th^ percentile (41). Similarly, other studies in adolescents reported that boys were more likely to report MetS factors, such as high blood pressure, elevated cholesterol, glucose, and triglyceride levels (42, 43). Although in some studies MetS patterns do not differ in both sexes (44), however, there are mechanisms that support evidence of sex differences (42, 43, 45, 46). In general, it has been found that men have higher visceral adipose tissue, intramyocellular and intrahepatic lipids than women, which could partly explain why they have a higher MetS (46). On the other hand, males tend to have higher blood pressure and cholesterol levels than women from puberty onward, which can also contribute to a higher cardiovascular risk (42, 43). In hormonal terms, it has been found that testosterone levels in men can negatively affect glucose metabolism and increase the risk of insulin resistance and type 2 diabetes, which are considered cardiometabolic complications (45).

Chronological age remains one of the strongest predictors of cardiometabolic events (47). In the present study, we found that participants who were 16 or older tended to report MetS; furthermore, an age ≥ 16 years was significantly associated with MetS. These findings are similar to the results reported in a recent study conducted in Peruvian adolescents where the highest proportion of those with MetS, measured by WHtR, were aged 15 to 17 years vs. 12 to 14 years (4). In addition, these results confirm the findings of research that measured waist circumference and WHtR in US children and adolescents (48). This study showed that the relative changes in WHtR increased with increasing age, and that the greatest relative change was observed in men and women between 18 and 19 years of age (48). Chronological age is an important determinant of health, since it coincides with the critical moments of increasing body fat and, therefore, of the development of diseases (49). In fact, as age increases, the risk of developing various chronic cardiometabolic diseases and conditions increases (48, 50). In addition, the physical and mental changes that occur with age can affect the quality of life and a person’s ability to perform daily activities (51). However, it is important to note that the premature onset of age-related diseases in younger people suggests a discrepancy between chronological and biological age, pointing out that chronological age is not always representative of true biological age, because several disease/morbidity factors may be related to biological age (50). Beyond this discrepancy, it is important to consider age as an important factor in assessing adolescent health and in planning long-term disease prevention and treatment strategies.

Global obesity and MetS, measured using WHtR, are two anthropometric factors that are associated with the onset and development of noncommunicable diseases (1). The measurement of both factors is particularly important in adolescents, since adolescence is a high-risk stage and one of the most critical periods of life, due to constant changes in lifestyle (52). Evidence for the association between global obesity and MetS has been demonstrated in both adolescents and the general population (4, 49, 53, 54). In our study, we found that those who had excess body weight were more likely to present MetS. These results are consistent with the findings of a study conducted in Spanish schoolchildren aged 6 to 15 years, showing a relationship between excess body weight and abdominal obesity, a metabolic risk factor (49). Similarly, the results of a recent study conducted in 506 adolescents aged 10 to 19 years of age from different schools in Brazil reported that normal weight obesity, which is defined as excess body fat in normal weight individuals, is associated with MetS, assessed through waist circumference (53). Some possible justification why obesity is related to increased waist circumference in adolescents is the fact that excess body fat accumulates predominantly in the abdominal region (1). It is worth mentioning that abdominal fat, also known as visceral fat, is metabolically active and can release pro-inflammatory fatty acids and adipokines, which contribute to the development of insulin resistance, dyslipidemia and other MetS factors (53, 54). Therefore, assessment of MetS measured through WHtR in obese and normal weight adolescents can be useful to identify those with a higher risk of developing cardiovascular and metabolic diseases.

Another relevant finding of this study is the fact that SNA is associated with MetS. This connection is especially relevant in the current times since the use of social networks has become a widespread and popular activity among adolescents (8). Although there is a paucity of research analyzing the relationship between SNA and MetS, our findings are in line with a recent study that evaluated the relationship between adolescents’ social networks and their health in adulthood suggesting that their position in their social network during adolescence has lasting implications for MetS in adulthood (10). It should be noted that most studies have focused on BMI, pointing to a possible relationship between SNA and excessive media use with an increased risk of developing obesity in children and adolescents (11–13, 55), which is related to cardiometabolic problems (1, 4). Therefore, it is important to highlight that excessive exposure to digital platforms could have negative effects on the cardiovascular health of this population group through the onset of obesity (56). This suggests that the effect of SNA on MetS could be mediated by obesity. Although it is important to note that structural equation modeling was not used in this study to explore these relationships in depth, our findings provide a clear picture of how cardiometabolic disease is influenced by SNA and obesity. This sets the groundwork for future research using mediation models, which will help to better understand these relationships. The mechanism for this association may be that excessive and sedentary use of social networks reduces the time that would be devoted to physical activities (57). Studies that have provided evidence to support this theory show that when the amount of time adolescents spend in front of screens is reduced, their level of physical activity increases (12). Another reason for this relationship is the fact that it has been suggested that the consumption of hypercaloric foods can increase in parallel with the time spent on the media and social networks (58, 59). This hypothesis is supported by research showing that high use of social networks among adolescents is associated with unhealthy dietary behaviors (60), and that energy intake in adolescents decreases when sedentary behaviors are reduced (59).

Finally, in this study, we found that anxiety symptoms are associated with MetS. Although little research has been done on the relationship between anxiety and metabolic risk factors in adolescents, however, a recent study has found a significant association between anxiety and some metabolic risk factors in this population group (18). Similarly, another study conducted in adolescents reported a relationship between anxiety symptoms and insulin resistance (19), which may lead to worsening metabolic outcomes in at-risk youth. Furthermore, studies have shown that anxiety symptoms are associated with an increased risk of cardiovascular disease and other metabolic problems in young adults (16, 17). Anxiety is a common affective disorder in children and adolescents, affecting approximately 1 in 12 children and 1 in 4 adolescents, and is one of the most common mental health problems in these populations (14). One of the possible reasons why anxiety symptoms are associated with MetS is that this disorder can contribute to increased intake of unhealthy foods and decreased physical activity (61). In particular, it is possible that people with anxiety are more likely to have a sedentary lifestyle, increasing the risk of obesity and cardiovascular disease (61). Furthermore, anxiety symptoms can increase the release of stress hormones, such as cortisol, which can promote abdominal fat gain and insulin resistance (19, 62).

### Limitations and future research

When interpreting the results of this study, certain limitations should be taken into account, which will benefit future lines of research. First, the study was a cross-sectional design; therefore, it does not allow establishing the possibility of causality, that is, it cannot be considered that having SNA, anxiety symptoms, or excess body weight can lead to an increase in WHtR; therefore, longitudinal studies that follow participants over time are needed to determine whether the initial presence of SNA, anxiety symptoms, or overweight predicts future increases in WHtR. Second, in relation to the anthropometric data, this is cross-sectional information, where a single measurement was taken for each student and there is no follow-up data to evaluate the evolution of weight and height over time. Therefore, the results presented are based on single measurements taken in different age groups. Considering this, we cannot evaluate how these anthropometric parameters evolve with age. However, in the specific case of WHtR, an advantage of using this index is that it does not appear to be age dependent at certain levels and therefore it may be possible to use a single cut-off value for all children (63). Nonetheless, it is important to point out that there is no consensus on a single WHtR cut-off point to predict the risk of MetS risk in adolescents (64). Given the lack of consensus on a single WHtR cut-off point for predicting the risk of MetS in adolescents, further studies are needed to explore and validate uniform criteria. This could include comparative analyses of different cut-off values in different adolescent populations to identify those that are most predictive of MetS risk. Third, data on SNA and anxiety symptoms were self-reported, which may lead to measurement errors. However, both instruments were validated in the Peruvian population. Therefore, future research on anxiety symptoms could be based on medical diagnoses rather than self-report alone. Medical diagnoses provide a more objective and detailed assessment of anxiety status by combining clinical observations, medical records, and, in some cases, psychometric tests administered by professionals (65). Finally, it is important to note that the inability to generalize the findings to a larger population, due to the type of sampling used and the number of participants involved, is an obvious limitation in the current study. Therefore, it is important to interpret the results with caution and within the specific context of the selected sample group. In addition, studies using probability sampling methods are suggested to verify and expand on the findings of the current study.

### Public health implications

Despite these limitations, we believe that the current study is of public health relevance due to its potential impact on the long-term health of adolescents. Adolescents are a vulnerable and growing population; therefore, habits and behaviors acquired at this stage can influence their health in adulthood. In addition, SNA, anxiety symptoms, and obesity are mental and physical health problems that have been increasing in the adolescent population in recent years. If these problems are related to an increased risk of MetS, they need to be addressed early and effectively. Therefore, it is important to conduct further research to confirm the relationship between these factors and to develop and implement preventive and treatment interventions targeting this vulnerable population. Furthermore, it is essential that health professionals, educators, and public health authorities inform and educate both adolescents and their parents about the importance of a healthy and balanced life, including responsible use of social networks, management of stress and anxiety, and maintaining a healthy weight.

## Conclusion

The findings of this cross-sectional study suggest that men were more likely to have MetS compared to females; furthermore, we found that a higher proportion of men had higher MetS. Furthermore, it is notable that participants 16 years or older tended to report a higher level of MetS; furthermore, an age ≥ 16 years was significantly associated with the risk of MetS. Similarly, excess body weight, SNA, and anxiety symptoms were associated with the risk of MetS. Given the impact of MetS on health, more efforts are needed to better understand the associated factors for the implementation of effective preventive and therapeutic interventions.

## Data availability statement

The raw data supporting the conclusions of this article will be made available by the authors, without undue reservation.

## Ethics statement

The studies involving humans were approved by Research Ethics Committee of the Universidad Señor de Sipán (Registration and reference number: 0085-17052022-CIEI). The studies were conducted in accordance with the local legislation and institutional requirements. Written informed consent for participation in this study was provided by the participants’ legal guardians/next of kin.

## Author contributions

JS: Conceptualization, Funding acquisition, Investigation, Methodology, Project administration, Supervision, Visualization, Writing – original draft, Writing – review & editing. SO-G: Conceptualization, Data curation, Funding acquisition, Investigation, Project administration, Resources, Writing – original draft. GL-T: Conceptualization, Methodology, Project administration, Resources, Visualization, Writing – original draft. IL-D-M: Conceptualization, Data curation, Funding acquisition, Writing – original draft. FB-C: Funding acquisition, Investigation, Methodology, Writing – original draft. EL-L: Conceptualization, Funding acquisition, Methodology, Project administration, Writing – review & editing. YC-M: Conceptualization, Data curation, Investigation, Project administration, Visualization, Writing – original draft, Writing – review & editing. AS-B: Investigation, Methodology, Validation, Visualization, Writing – review & editing. CR-V: Formal Analysis, Investigation, Methodology, Supervision, Visualization, Writing – review & editing.
